# 

**Published:** 1873-08-01

**Authors:** 


					﻿THE
Medical Examiner.
A Semi-Monthly lournal of Medical Sciences.
No. 15.
CHICAGO. AUGUST 1, 1873.
Vol. XIV.
TERMS.—Yearly Subscriptions, Three Dollars, in advance. Single Numbers Fifteen Cents.
All Communications should be addressed to Publishers Medical Examiner, 792 Wabash Av., Chicago.
				

